# *Catabacter hongkongensis* Bacteremia with Fatal Septic Shock

**DOI:** 10.3201/eid1707.101773

**Published:** 2011-07

**Authors:** Antoine Elsendoorn, René Robert, Agnès Culos, France Roblot, Christophe Burucoa

**Affiliations:** Author affiliations: Centre Hospitalier Universitaire de Poitiers, Poitiers, France (A. Elsendoorn, R. Robert, A. Culos, F. Roblot, C. Burucoa); University of Poitiers, Poitiers (A. Elsendoorn, R. Robert, F. Roblot, C. Burucoa)

**Keywords:** bacteria, septic shock, bacteremia, Catabacter hongkongensis, letter

**To the Editor:** *Catabacter hongkongensis* is a newly described anaerobic agent that is likely an intrinsic component of normal gut flora; it was first reported by Lau et al. in 2007 (*1*). We report a fatal case of infection caused by *C. hongkongensis* that was identified by 16S rRNA sequence.

A man 52 years of age was admitted to an intensive care unit in France for septic shock. He was a retired service member, smoker, and alcohol drinker. He had a history of hypertension but no previously known gastrointestinal disease. He sought treatment for acute abdominal pain and diarrhea of several hours’ duration. On admission, he had persistent abdominal pain with generalized abdominal distension, arterial hypotension, and hypoxemia but was not febrile. Two sets of anaerobic and aerobic blood cultures were performed at a 1-hour interval, and empiric treatment with amoxicillin/clavulanic acid and gentamicin was started. Biochemical screening showed severe metabolic acidosis, acute renal insufficiency, and systemic inflammatory response syndrome. An abdominal radiograph revealed massive pneumoperitonitis. Laparotomy showed multiple lesions and intestinal perforation at the ascending and first part of the transverse colon, with a large amount of purulent fluid in the peritoneal cavity. A complete colectomy was performed, with rectum closure and end ileostomy. Despite fluid resuscitation and catecholamine infusions, hemodynamic instability worsened rapidly and led to the patient’s death.

Microbiological analysis of abdominal fluid revealed the presence of *Pseudomonas aeruginosa*, *Escherichia coli*, and *Enterococcus* spp., but no anaerobic agent. On day 3 postincubation, 1 of the 2 anaerobic blood cultures grew a motile gram-positive bacillus, which grew only in strictly anaerobic conditions. Phenotypic analysis showed catalase production but not indole positivity or nitrate reduction. Standard phenotypic tests were performed with the rapid ID 32A and api20A strips (bioMérieux, Marcy l’Etoile, France). The numeric profiles obtained were 0002000010 and 4030121, respectively. The bacteria produced acid from arabinose, glucose, mannose, and xylose, was negative for glycerol fermentation and leucine arylamidase and positive for rhamnose fermentation. Despite these results, phenotypic tests failed to identify the isolate. Antimicrobial drug susceptibility was determined by disk diffusion method and Etest for MICs. The isolate showed susceptibility to metronidazole (MIC <0.016 µg/mL), vancomycin, and colistin (MIC <0.016 µg/mL) and resistance to penicillin (MIC 2 µg/mL), gentamicin, netilmycin, kanamycin, amikacin, and cefotaxime (MIC >32 µg/mL) according to Eucast clinical breakpoints (www.eucast.org). No other bacteria were isolated in the blood cultures.

Genetic analysis was performed by 16S rRNA gene sequencing of a 1,265-bp fragment by using DG74 and RDRO80 primers (*2*). The nucleotide sequence obtained was compared with known sequences in GenBank by multiple sequence alignment using the ClustalW program (*3*). It was 100% identical to *C. hongkongensis* (GenBank accession no. AY574991).

The first 4 case-patients with *C. hongkongensis* infection were described by Lau et al. in 2007 (*1*). Two of these patients lived in Hong Kong and the 2 others in Canada. As in our case, only 1 patient died. Since there was a high degree of phenotypic and genetic difference with other anaerobic agents, the authors proposed a new genus and species and affiliation with a new family, *Catabacteriacae.* The 2 isolates from Canada differed from the 2 others by being negative for glycerol fermentation and positive for rhamnose fermentation and leucine arylamidase, similar to our case, except for leucine arylamidase, which in our case was negative. In the previously reported cases, *C. hongkongensis* was susceptible to metronidazole, vancomycin, and kanamycin; variably susceptible to penicillin (MICs 0.5–4.0 µg/mL); and resistant to colistin and cefotaxime (*1*).

Whether *C. hongkongensis* belongs to the intestinal flora, as do *Bifidobacterium*, *Eggerthella*, *Eubacterium*, and *Lactobacillus* spp., remains undetermined. Codony et al. recently investigated by real-time PCR the presence of *Catabacteriaceae* in 29 water samples in the vicinity of Barcelona, Spain. Four samples were positive, demonstrating presence of this organism in the European environment and its probable enteric origin (*4*).

Because our patient sought treatment with severe infection associated with isolation of other pathogenic bacteria, whether blood infection by *C. hongkongensis* may be responsible for such a fatal outcome is unknown. Nevertheless, we can exclude sample contamination by this anaerobic bacteria for the 2 following reasons. First, anaerobic contaminants are rare in blood cultures and generally involve *Propionibacterium acnes*. Furthermore, the rapid growth of the present isolate in blood cultures within 3 days suggested a relatively high bacterial load in the blood sample.

Our report confirms that *C. hongkongensis* can be found in blood culture associated with gastrointestinal disease and may reflect intestinal perforation. Identification may be difficult. Isolation of motile gram-positive anaerobic bacillus together with catalase positivity should lead to suspicion of *C. hongkongensis* in clinical laboratories. Full identification of this pathogen requires 16S sequencing. Environmental reports have demonstrated the presence of this organism in human wastewater in Europe, which suggests that it may be universally present as part of the normal human gastrointestinal flora.
